# Imaging of the spectrum of bony injuries in the diabetic foot: a case series with emphasis on non-Charcot fractures

**DOI:** 10.1259/bjrcr.20170026

**Published:** 2017-08-04

**Authors:** Marcela Mautone, Parm Naidoo, Kevin Zhou

**Affiliations:** ^1^Diagnostic Imaging Department, Monash Health, Melbourne, Australia; ^2^Monash University, Melbourne, Australia; ^3^University of Melbourne, Melbourne, Australia

## Abstract

Diabetes mellitus is associated with an increased risk of lower limb injuries. Peripheral neuropathy, often associated with diabetes, has been demonstrated to increase the risk of fracture almost two-fold and is associated with complications related to fracture healing. Detection of neuropathy-related foot injury is frequently delayed owing to the paucity of symptoms and low degree of suspicion by the clinician. Early recognition of fracture or bone injury and appropriate treatment are critical in preventing debilitating foot deformity and disability. Therefore, the astute radiologist cognizant of these potential injuries plays an essential role in early diagnosis of bony injuries in the diabetic foot. We present a series of radiological images that depict a range of osseous injuries in the diabetic foot and emphasize the role of the radiologist in early recognition of these abnormalities.

## Background

Diabetes mellitus is associated with an increased risk of lower limb fractures.^1^ Peripheral neuropathy, often associated with diabetes, has been demonstrated to increase the risk of fracture almost two-fold and is associated with complications related to fracture healing.^1,2^ An estimated 20 to 30 million people are affected by diabetic neuropathy worldwide; however, the condition often remains undiagnosed until a painless foot injury occurs.^3,4^

Charcot neuropathic osteoarthropathy (CN) is characterized by bone and joint destruction of the foot and ankle associated with diabetic peripheral neuropathy. The onset of Charcot arthropathy is commonly preceded by an undetected injury that initiates an uncontrolled inflammatory process.^5^ Joint destruction and spontaneous fractures are known consequences of CN. However, non-CN related bone remodelling, chronic bone injury and fractures in the diabetic foot prior to the development of neuroarthropathy have been largely neglected in the extant literature.

Repeated microtrauma in the insensate foot is accepted as a risk factor for the development of injury in diabetic patients.^6^ In addition, patients with diabetes with neuropathy tend to have higher plantar pressures than non-diabetic individuals or patients with diabetes without neuropathy, further increasing the risk of bone fractures.^7^ Boulton et al^7^ have posited that the cause of the higher plantar pressures is related to impairment of muscle function secondary to neuropathy. Reduction in the thickness of the submetatarsal fat pads in the diabetic patient may also play a role.^7^ Furthermore, in an obese diabetic patient, increased loading further elevates the risk of foot injury.^8^

A study by Poll and Chantelau demonstrated that occult traumatic bone injuries are present in up to 80% of asymptomatic feet in diabetic patients with peripheral sensory nerve dysfunction.^8^ With continued mechanical load and diminished sensation, an initial minor injury may progress to irreversible foot deformity and subsequent disability.

Detection of neuropathy-related foot injury is frequently delayed owing to paucity of symptoms and low degree of suspicion by the clinician. Early recognition of fracture or bone injury and appropriate treatment in patients with diabetes with decreased sensation is critical in preventing debilitating foot deformity and disability. Therefore, the astute radiologist cognizant of these potential injuries plays an essential role in early diagnosis of bony injuries in the diabetic foot.

We present a series of radiological images that depict a range of osseous injuries in the diabetic foot and highlight the role of the radiologist in early recognition of these abnormalities. Where appropriate, we postulate potential mechanisms in individual cases, several of which manifest on imaging studies and which should be emphasized by the reporting radiologist.

## Stress fractures

### Case 1

**Figure 1.**
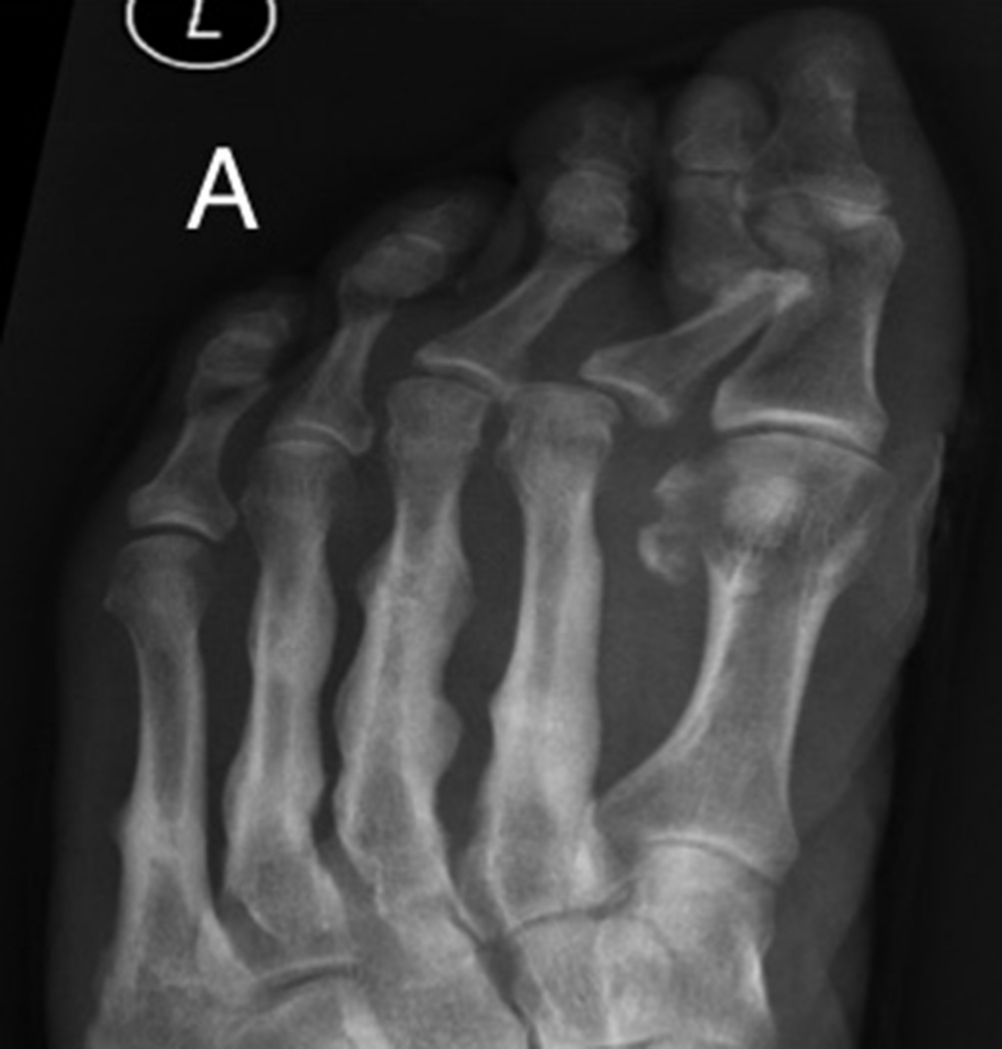
Radiograph of the left foot demonstrates callus formation at the diaphysis of the second, third, fourth and fifth metatarsals, consistent with stress fractures.

A 58-year-old male with Type 2 diabetes mellitus and peripheral neuropathy, presented with left foot swelling and minimal pain. There was no history of trauma. Radiograph (Figure 1) demonstrates multiple healing stress fractures involving the diaphysis of the second, third, fourth and fifth metatarsals.

## Discussion

Bone stress injuries are usually acquired during voluntary overuse activities by athletes, most commonly affecting the distal lower limb.^9^ Stress injuries may also develop during everyday load-bearing activities due to cyclic overuse. Such injuries are of concern in patients with diabetes with deficient pain sensation from diabetic polyneuropathy as foot overuse may not be perceived. With continued mechanical load and absence of sensation, an initial stress injury may progress to a complete fracture. Early diagnosis and immediate treatment of stress fractures through offloading and immobilization may decrease the likelihood of progression to stage 1 Charcot neuropathic osteoarthropathy of bone dissolution or complete fracture, and prevent irreversible foot deformities and amputation.^9,10^

Conventional radiography is the primary imaging modality for diagnosing stress fractures; however, early bone stress injuries may not be displayed.^11^ The most sensitive modality for diagnosing stress injuries is MRI. MRI provides valuable information about the surrounding soft tissues and is very sensitive in detection of bone bruising, bone marrow oedema and microfractures associated with chronic stress responses. Bone marrow oedema represents an early sign of stress-related injury to bone; however, this is a non-specific finding that can also be present in osteomyelitis, tumour and bone bruise (contusion).^12^ Infection and malignancy usually demonstrate soft tissue involvement.^12 ^

## Key learning point

1. Bone stress fractures are rarely diagnosed in patients with diabetes with neuropathy because of their atypical presentation with foot oedema rather than pain, exacerbated by loadbearing.^9^ Foot swelling must be investigated promptly in patients with neuropathy and stress fracture must be considered in the differential diagnosis.
2. Plain radiography is the primary modality of investigation followed by MRI if a stress fracture remains suspected.
3. Diffuse marrow oedema within one or more metatarsals associated with circumferential cortical thickening in the absence of focal cortical destruction and a penetrating infected ulcer is highly specific for a chronic stress injury.

## Avascular necrosis

### Case 2

**Figure 2.**
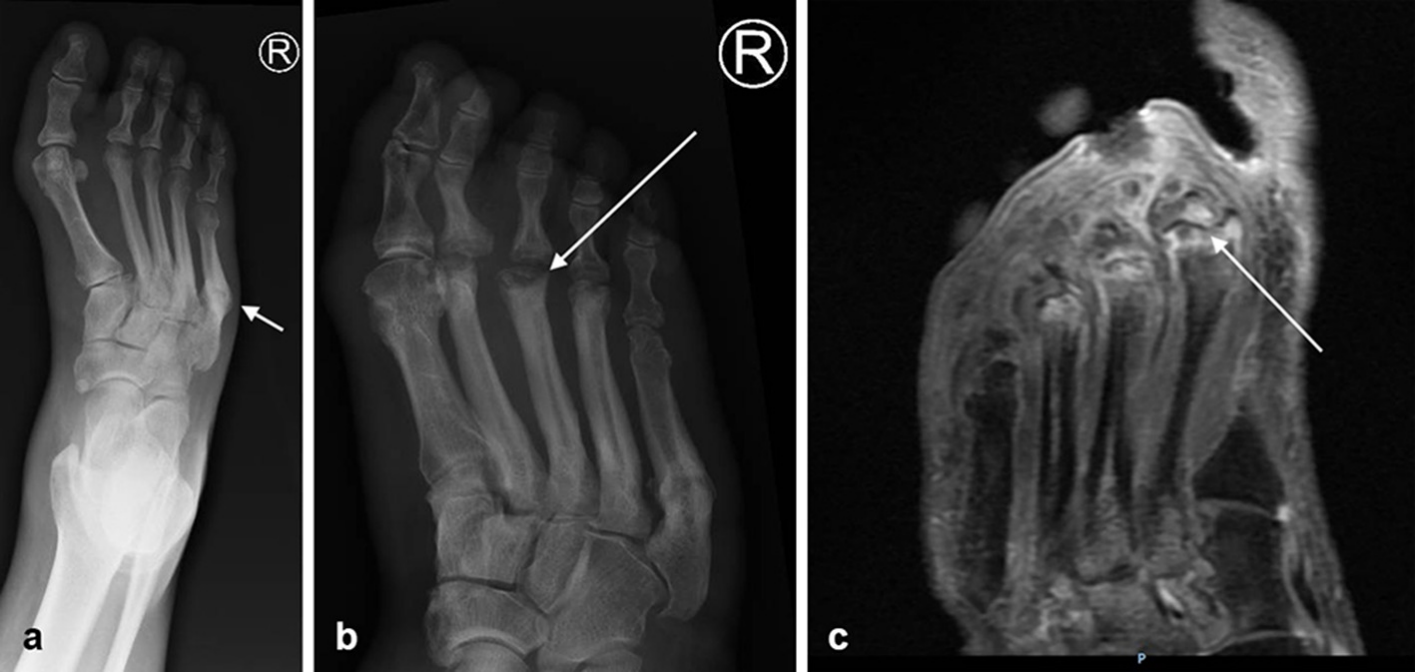
(a) Right foot radiograph 6 months prior to presentation demonstrates an ununited fracture of the proximal shaft of the fifth metatarsal (arrow). Note exuberant callus formation and sclerotic bone margins, likely representing a post fracture pseudarthrosis. (b,c) Right foot radiograph (b) of the same patient performed for investigation of new foot swelling, followed by MRI (c). Radiograph (b) demonstrates collapse and sclerosis of the second, third (arrow) and fourth metatarsal heads. Note associated cortical thickening of the metaphyses and diaphyses. Axial post gadolinium fat-suppressed *T*_1_ sequence (c) demonstrates increased signal in the third, fourth and fifth metatarsal heads and bone marrow oedema with no significant soft tissue involvement, confirming avascular necrosis.

A 46 year-old female with a history of poorly controlled Type 1 diabetes mellitus and diabetic peripheral neuropathy presented with painless foot swelling. Radiograph performed 6 months prior to presentation (Figure 2a) shows an ununited fracture of the proximal shaft of the fifth metatarsal. No specific treatment for the ununited fracture was documented. Six months later, a radiograph (Figure 2b) performed for investigation of painless foot swelling demonstrates changes consistent with avascular necrosis of the heads of the second, third and fourth metatarsals. MRI (Figure 2c) confirmed the findings. In the setting of diabetic peripheral neuropathy, we postulate that the development of avascular necrosis is most likely secondary to altered biomechanics due to the previous fifth metatarsal fracture and is a result of increased force exerted on the metatarsal heads, poor bone quality and microvascular ischaemia.

### Case 3

**Figure 3.**
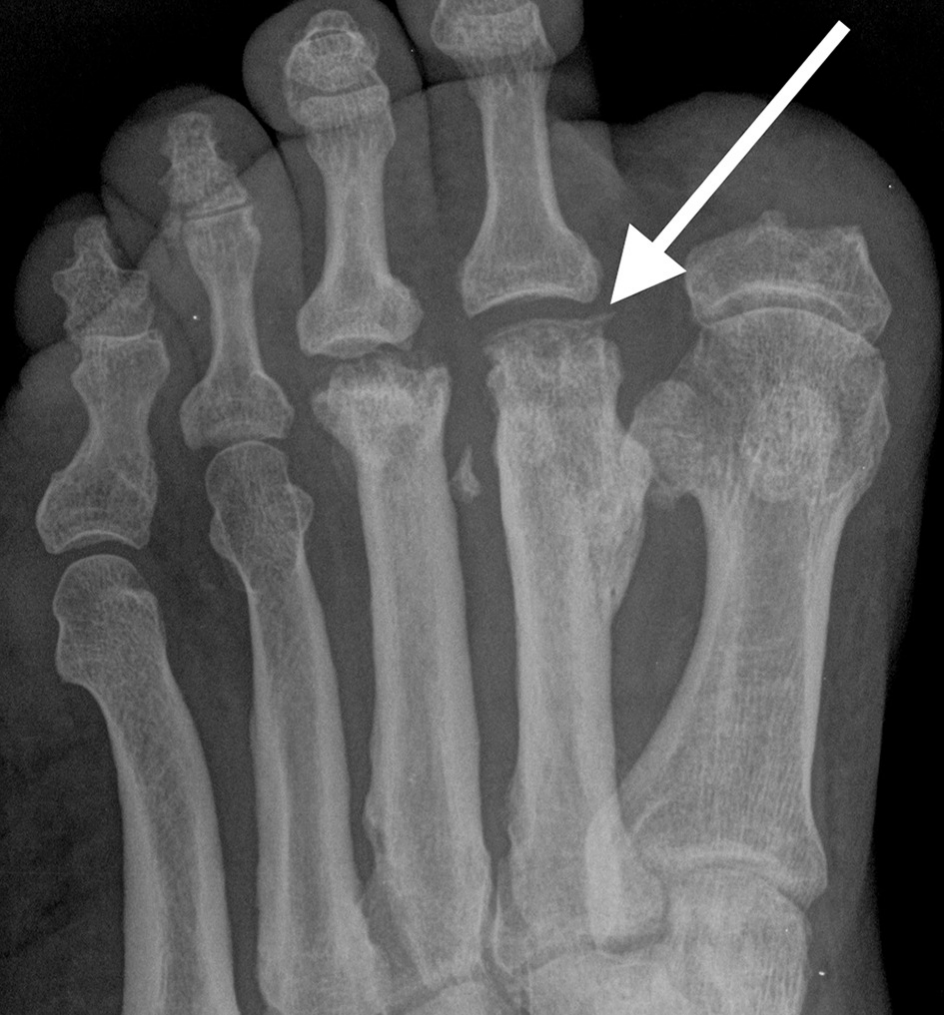
Radiopraph of the left foot demonstrates flattening of the heads of the second (arrow) and third metatarsals, consistent with established avascular necrosis. Note associated diffuse cortical thickening along the metaphyses and diaphysis of the involved metatarsals, commonly seen in avascular necrosis.

A 53-year-old male with Type 1 diabetes, peripheral neuropathy and previous partial amputation of the proximal phalanx of the left great toe presented with a non-healing, minimally painful ulcer at the stump of the left first toe. Radiograph of the left foot (Figure 3) demonstrates established avascular necrosis involving the heads of the left second and third metatarsals. Amputation of the proximal phalanx of the first toe with resultant alteration in biomechanics is the likely initiating factor for osteonecrosis of the metatarsal heads.

## Discussion

Avascular necrosis of the pedal bones is a recognized, albeit uncommon, complication of diabetes mellitus. The talus, navicular, first and second metatarsal heads are the most common sites of bone infarction.^13^ Freiberg’s disease is an ischaemic necrosis of the metatarsal head, with the second metatarsal head being most commonly involved as it is subjected to the greatest reactive ground forces during ambulation.^14^ Its presentation is predominantly in late childhood or adolescence and has been associated most often with females. Diabetes mellitus may be a predisposing factor in the development of the disease in adults. In diabetic patients, atrophy of intrinsic small foot muscles secondary to diabetic neuropathy may play a role in the development of osteonecrosis.^14^ The atrophic muscles cause the toes to be drawn into a position called “claw toes.” As a result, the toes no longer participate in the distribution of load on the foot and weight bearing is then shifted to the metatarsal heads, which are less protected by atrophic muscles.^14^ The stress on the metatarsal heads leads to microfractures, vascular injury and subsequent avascular necrosis.

Early radiographic findings include subtle flattening of the metatarsal head, areas of increased radiodensity or mixed sclerosis and lucency (mottling). Later findings include osteochondral fragmentation, with progressive flattening and sclerosis of the metatarsal head, and cortical thickening of the metaphysis and diaphysis.^14^

MRI can identify early avascular necrosis prior to CT or radiographic changes, or indeed clinical symptoms, particularly in the neuropathic lower limb. The infarcted medullary bone of inhomogeneous signal intensity is classically surrounded by an inner hyperintense and outer hypointense “double line” on *T*_2_ weighted images, which represents vascular granulomatous tissue underneath sclerotic bone.^15–18^ However, the double-line sign is frequently absent in small pedal bones, in which poorly defined areas of *T*_1_ hypointensity may be seen without contrast enhancement.^19^

Based on our experience, MRI frequently shows that chronic stress-related medullary oedema is accompanied by circumferential cortical thickening, typically seen in the diaphysis and metaphysis of the metatarsals; however, this has also been described in association with Freiberg’s disease.^14^

## Key learning point

Altered biomechanics related to obesity, previous toe amputation or foot ulcer as well as high plantar pressures and muscle dysfunction predispose patients with diabetes to avascular necrosis.

## Bone remodelling

### Case 4

**Figure 4.**
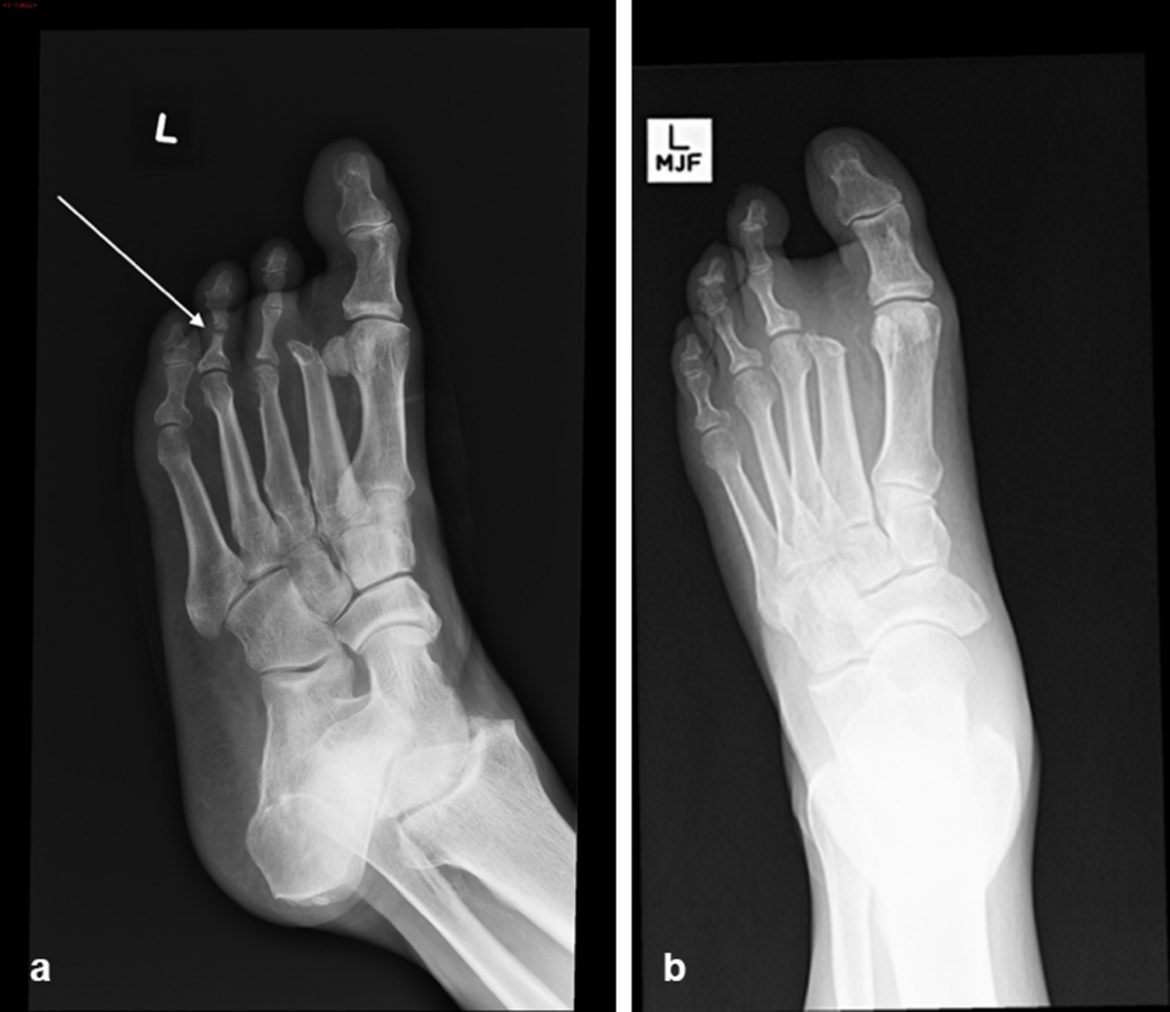
(a) Oblique radiograph of the left foot demonstrates previous amputation of the second toe and an ununited fracture of the head of the proximal phalanx of the fourth toe, probably secondary to previous trauma in an insensate foot. Furthermore, there is “pencil point” deformity of the neck of the proximal phalanx of the fourth toe (arrow). Cortical irregularity at the head of the third metatarsal raises suspicion of osteomyelitis. (b) Left foot radiograph of the same patient performed 5 years earlier, shortly after amputation of the second metatarsal, demonstrates normal appearance of the phalanges of the fourth toe.

A 65-year-old male with Type 2 diabetes mellitus and peripheral neuropathy presented with a painless ulcer in the third toe. Radiograph performed on presentation (Figure 4a) shows an ununited fracture of the head of the proximal phalanx of the fourth toe, consistent with previous trauma in an insensate foot. “Pencil point” deformity of the neck of the proximal phalanx of the fourth toe is compatible with bone remodelling, most likely related to chronic microtrauma associated with profound sensory neuropathy and altered biomechanics secondary to previous amputation. A radiograph (Figure 4b) performed 5 years earlier shown for comparison demonstrates normal appearance of the proximal phalanx of the fourth toe.

### Case 5

**Figure 5.**
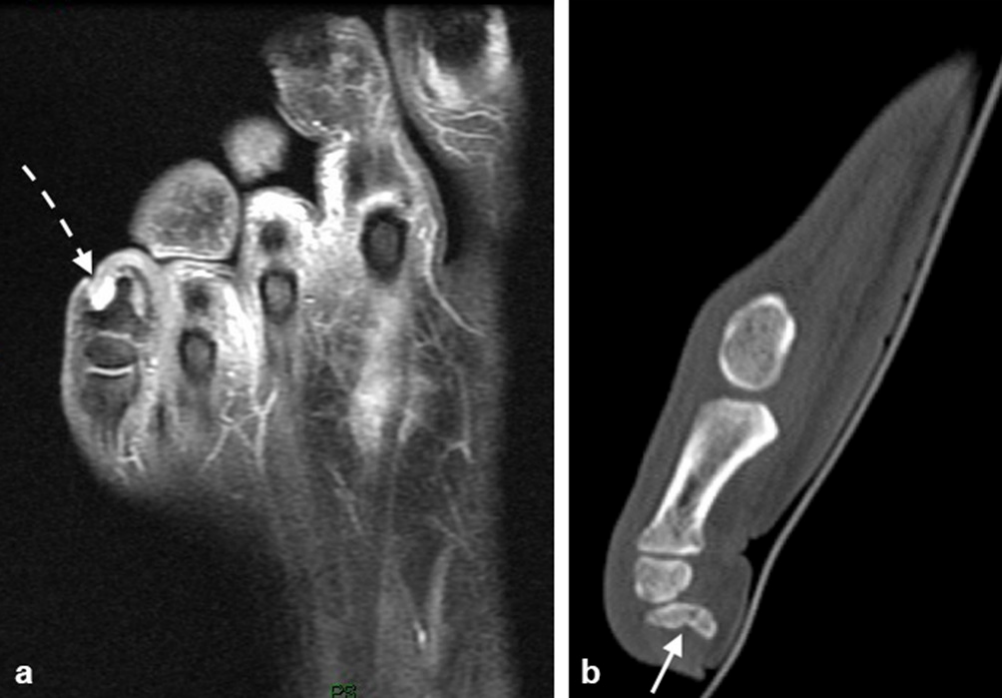
MRI axial post gadolinium fat suppressed *T*_1_ sequence (a) and sagittal reformatted CT (b) demonstrate resorptive remodelling of the distal phalanx of the fifth toe (arrow). Surrounding soft tissue enhancement (dashed arrow) is noted on the MRI.

A 52-year-old male patient with a history of diabetes mellitus and peripheral neuropathy presented with fifth toe swelling. MRI (Figure 5a) and CT (Figure 5b) demonstrate remodelling of the distal phalanx of the fifth toe. The MR appearances are suggestive of chronic microtrauma in the context of diabetic neuropathy with remodelling of the bone with periosteal and soft tissue reaction.

## Discussion

Acro-osteolysis refers to a slow, progressive resorption of the distal phalanges of hands and feet.^20^ There are two forms that differ in their distribution and associations. The more common form affects the terminal tufts of the phalanges, while the other form is known as band acro-osteolysis and transversely affects the distal shaft.^20,21^

When affecting the terminal tufts, bone resorption leads to osteosclerosis and foreshortening of the distal phalanges.^22^ This is more strongly associated with a rheumatological cause such as systemic sclerosis.^23^ Band acro-osteolysis is typified by a sharply demarcated osteolysis of the mid-shaft of the distal phalanges and is strongly associated with polyvinyl chloride exposure and Hadju–Cheney syndrome.^21,24^

Acro-osteolysis is associated with a wide range of disease processes including metabolic disorders such as hyperparathyroidism and diabetes mellitus, rheumatological conditions, repetitive trauma, polyvinyl chloride exposure and a number of genetic conditions.^20,22,23^ While the exact pathogenesis is uncertain, it has been inferred from the known causes to involve microvascular ischaemia and severe sensory neuropathy.^20^ The established deformity is well demonstrated on plain radiography; however, MRI may show earlier changes of the process in evolution (as shown in case 5).

## Key learning point

Acro-osteolysis refers to progressive bone resorption affecting the distal phalanges of the hands and feet. It may occur in patients with diabetes with severe sensory neuropathy associated with repetitive trauma.

## Calcaneal fractures

### Case 6

**Figure 6.**
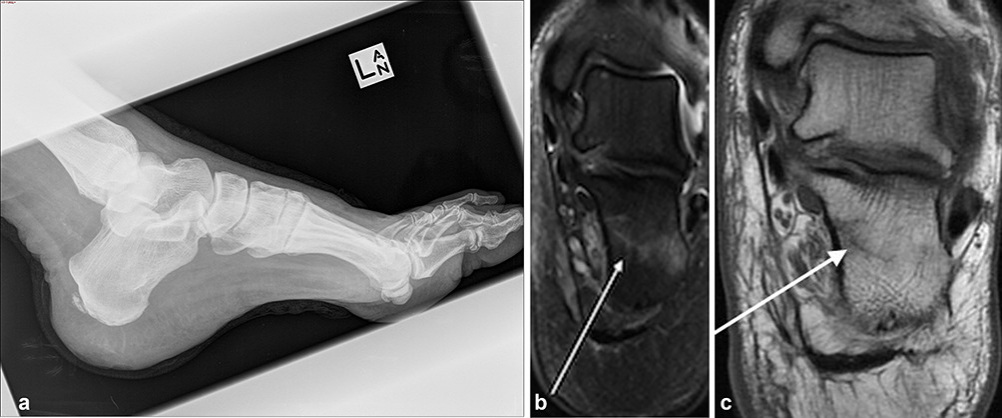
(a) Lateral radiograph of the left foot demonstrates a posterior calcaneal spur, but otherwise normal appearance of the calcaneum. (b,c) MRI coronal *T*_2_ fat suppressed sequence (b) demonstrates signal hyperintensity in the anterior calcaneus, which extends from the medial to the lateral cortex, with corresponding low signal on coronal *T*_1_ sequence (c). Findings are consistent with a non-displaced fracture. A soft tissue defect at the heel (not shown) does not extend to the calcaneal cortex. Remaining calcaneal signal (not shown) is normal with no evidence of osteomyelitis.

A 62-year-old male with a history of Type 2 diabetes was admitted with a chronic non-healing left calcaneal ulcer. Radiograph of the left calcaneus (Figure 6a) was unremarkable. MRI (Figure 6b,c) reveals a non-displaced insufficiency fracture of the calcaneus with no evidence of osteomyelitis.

### Case 7

**Figure 7.**
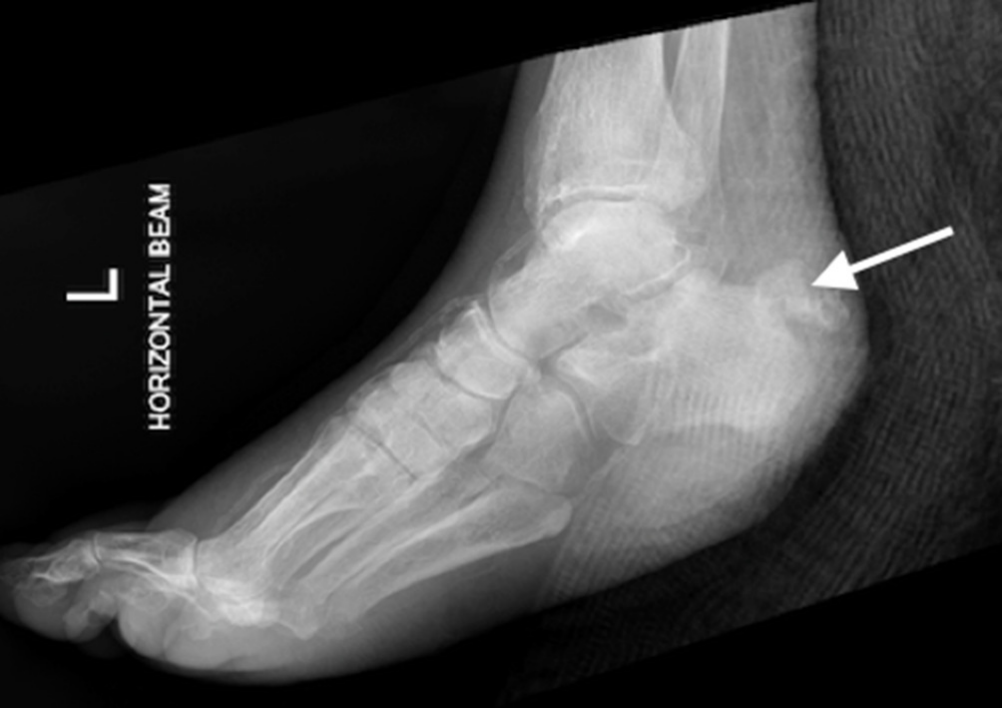
Radiograph of the left foot demonstrates a dressing overlying a heel ulcer and a wedge-shaped calcaneal avulsion fracture (arrow) at the posterior calcaneum.

A 73-year-old male with a history of Type 2 diabetes mellitus presented with a heel ulcer. Radiograph of the left foot (Figure 7) reveals a wedge-shaped avulsion fracture at the posterior calcaneus.

## Discussion

Atraumatic calcaneal fractures are characteristically seen in the diabetic foot and may result from peripheral neuropathy, gait abnormalities, decreased bone density, altered vascularity and ulceration with spread of infection. Peripheral neuropathy results in decreased proprioception and pain sensation with redistribution of the weight and consequent bone injury. Delay in recognizing bone injury in the neuropathic diabetic foot leads to marked alteration in gait, which in turn may contribute to an increased load on the calcaneus and/or Achilles tendon during walking.^25^ While neuropathy is a central feature for atraumatic calcaneal fractures, a combination of factors is usually present.

Calcaneal fractures in the absence of trauma may present as (1) a cleavage or wedge-shaped fracture extending from the calcaneal tubercle; (2) a mid-calcaneal compression fracture; (3) a superiorly displaced extra-articular posterior calcaneal avulsion.^13^ The wedge-shaped or cleavage type is typical in patients with a heel ulcer with weakened bone due to osteomyelitis and excessive traction from the Achilles tendon during ambulation (see case 7).^25^

A study of calcaneal fractures in 13 patients with diabetes demonstrated osseous union in the majority of patients. However, calcaneal deformity following an atraumatic fracture was seen in approximately 50% of the patients.^25^ Deformity may occur as a result of mechanical alterations in the foot and ankle, which may be due to accelerated CN in the foot and may predispose the patients to subsequent skin ulceration.^25^

## Key learning point

1. Calcaneal fractures can occur in patients with diabetes as a result of peripheral neuropathy, gait abnormalities, decreased bone density, altered vascularity and ulceration with spread of infection.
2. Plain radiography is the primary modality of investigation followed by MRI if a calcaneal fracture remains suspected.

## Mueller–Weiss syndrome

**Figure 8.**
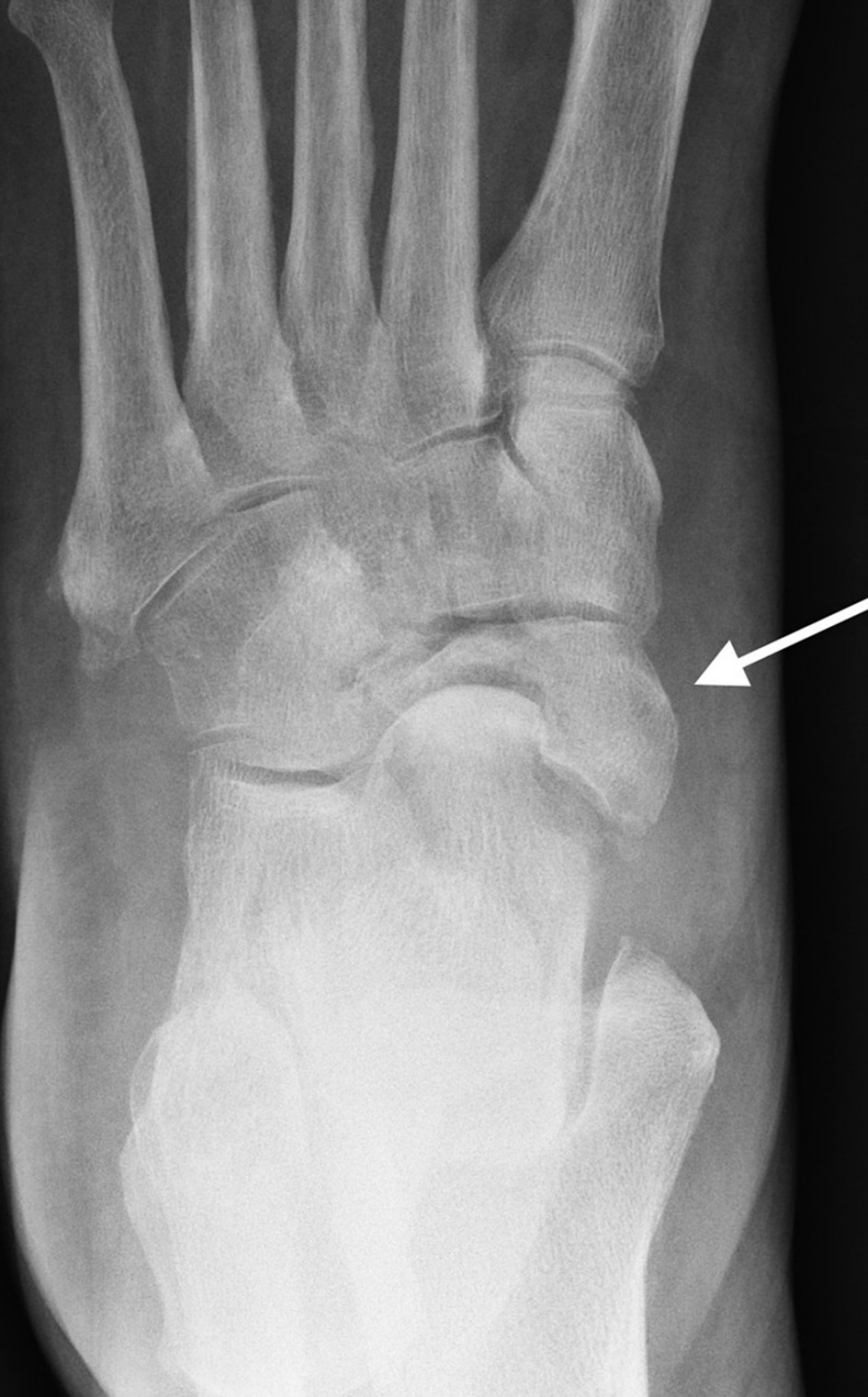
Radiograph of the left foot of a 63-year-old female demonstrates isolated destruction of the navicular (arrow) with remaining tarsal bones demonstrated to be intact. Features are consistent with established navicular osteonecrosis (Mueller–Weiss syndrome).

Case 8: A 63-year-old female with a history of Type 2 diabetes mellitus presented with left foot swelling for several weeks with vague mid-foot pain. No history of trauma was reported, nor was there evidence of peripheral neuropathy. Radiograph of the left foot (Figure 8) demonstrates isolated collapse of the navicular with marked sclerosis, consistent with osteonecrosis (Mueller–Weiss syndrome).

## Discussion

Mueller–Weiss syndrome is a rare condition that refers to spontaneous, adult-onset navicular osteonecrosis.^26^ It is generally bilateral and most often presents between 40 and 60 years of age with a female preponderance.^27^ The exact pathogenesis is not fully understood but the most accepted theory is of a multifactorial combination of chronic traumatic loading and ischaemia on a background of a suboptimally ossified bone.^26,28^ Trauma has also been postulated as a possible aetiology. In fact, appearances may be similar to a missed stress fracture.^29^ The syndrome is heavily associated with vascular risk factors such as diabetes mellitus, smoking and metabolic syndrome.^30^

The clinical presentation in Mueller–Weiss syndrome is non-specific with chronic mid-foot and hind foot pain, swelling and tenderness being the typical findings.^26,31^ More severe cases may develop a pes planovarus and pes planus as the navicular collapses and the medial longitudinal arch is consequently flattened.

Plain radiography is the investigation of choice; however, MRI may show early bone marrow changes and a pathognomonic high/low signal “double line” depicting the difference between live and dead bone.^32^ Isotope bone scans demonstrate increased tracer uptake and CT can often show sclerosis before the radiographic changes.

Plain radiograph of avascular necrosis may demonstrate the following progressive stages:

I, Diffuse osteoporosis; II, cysts with sclerotic margins; III, a subchondral area of lucency; IV, bone sclerosis; V, bone collapse and remodelling.^33 ^ Based on our experience, patients present in the later stages.

The most common findings are related to destruction of the lateral half of the navicular.^26^ It gradually becomes compressed and sclerotic with resulting peritalar subluxation of the medial aspect. This creates a “comma-shaped” navicular.^32^ Eventually, the lateral navicular will fragment, which is best appreciated on CT imaging, and neoarticulations between the talus and cuneiform bones can occur.

The management of Mueller–Weiss syndrome is initially based on offloading, orthotics, analgesia and lifestyle changes, which can effectively improve pain and function.^32,34^ If conservative methods fail, then surgical management is indicated. Mid-foot fusion is the operation of choice, generally across the talonavicular and naviculocuneiform joints, but there is no strong consensus on the exact techniques to be used.^34^ If bone union is achieved, there may be significant benefit to function and improvement in pain.

## Learning points

1. Navicular osteonecrosis can present as swelling and foot deformity in association with diabetic peripheral neuropathy. The condition shares similar clinical features with Charcot neuroarthropathy^31^ and appearances may be similar to a missed stress fracture.^34^
2. Changes in navicular bone density may be the only indication of osteonecrosis on radiographs.
3. When the diagnosis is in doubt, radionuclide scans and MRI may detect osteonecrosis earlier. MRI shows early bone marrow changes and a pathognomonic high/low signal “double line” depicting the difference between live and dead bone.

## Conclusions

Diagnosis of bone injuries is often delayed in patients with diabetes, especially in the presence of neuropathy where the presenting complaint is commonly foot oedema exacerbated by load bearing rather than pain.^9^ A fracture or atypical bone injury must be considered in the differential diagnosis of foot and ankle swelling and investigation must be initiated immediately. In our experience, the appreciation of a number of radiographic abnormalities related to the diabetic foot helps guide management to mitigate the risk of permanent deformity. The radiologist therefore plays an essential role in early diagnosis, and indeed, in prevention of bone fractures in patients with diabetes.
